# 2-(3-Chloro-5,6-diphenyl-2,5-dihydro-1,2,4-triazin-5-yl)-2-methyl­propane­nitrile

**DOI:** 10.1107/S1600536812023252

**Published:** 2012-05-31

**Authors:** Ewa Wolińska, Zbigniew Karczmarzyk, Andrzej Rykowski, Zofia Urbańczyk-Lipkowska, Przemysław Kalicki

**Affiliations:** a Department of Chemistry, Siedlce University, ul. 3 Maja 54, 08-110 Siedlce, Poland; b Institute of Organic Chemistry, Polish Academy of Sciences, ul. Kasprzaka 44/52, 01-224 Warsaw 42, POB 58, Poland

## Abstract

The title compound, C_19_H_17_ClN_4_, was obtained from the reaction of 3-chloro-5,6-diphenyl-1,2,4-triazine with isobutyronitrile in the presence of lithium diisopropyl­amide as an unexpected product of covalent addition of isobutyronitrile carbanion to the C-5 atom of the 1,2,4-triazine ring. The 2,5-dihydro-1,2,4-triazine ring is essentially planar (r.m.s. deviation = 0.0059 Å) and the 5- and 6-phenyl substituents are inclined to its mean plane with dihedral angles of 89.97 (4) and 55.52 (5)°, respectively. Intra­molecular C—H⋯N inter­actions occur. In the crystal, mol­ecules related by a *c*-glide plane are linked into zigzag chains along [001] by N—H⋯N hydrogen bonds.

## Related literature

For background information, see: Hargaden & Guiry (2009 ▶); Konno *et al.* (1987 ▶); Rykowski *et al.* (2000 ▶). For the synthesis, see: Coeffard *et al.* (2009 ▶); Fujisawa *et al.* (1995 ▶). For a related structure, see: Ayato *et al.* (1981 ▶).
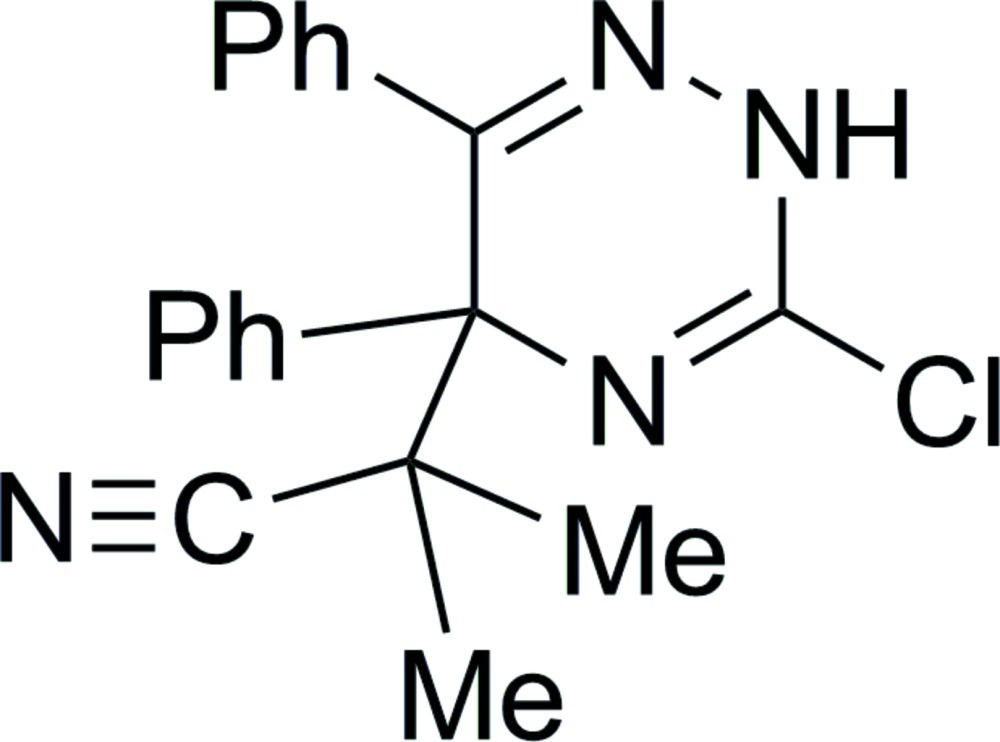


## Experimental

### 

#### Crystal data

C_19_H_17_ClN_4_
*M* *_r_* = 336.82
Monoclinic, 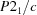

*a* = 8.2422 (1) Å
*b* = 13.9124 (2) Å
*c* = 15.4685 (3) Å
β = 93.855 (1)°
*V* = 1769.74 (5) Å^3^
*Z* = 4
Cu *K*α radiation
μ = 1.96 mm^−1^
*T* = 293 K
0.44 × 0.23 × 0.11 mm

#### Data collection

Bruker SMART APEXII CCD diffractometer
Absorption correction: multi-scan (*SADABS*; Bruker, 2005 ▶) *T*_min_ = 0.817, *T*_max_ = 1.000
20671 measured reflections
3198 independent reflections
2957 reflections with *I* > 2σ(*I*)
*R*_int_ = 0.037

#### Refinement

*R*[*F* ^2^ > 2σ(*F* ^2^)] = 0.037
*wR*(*F* ^2^) = 0.110
*S* = 1.03
3198 reflections
221 parameters
H atoms treated by a mixture of independent and constrained refinement
Δρ_max_ = 0.21 e Å^−3^
Δρ_min_ = −0.28 e Å^−3^

### 

Data collection: *APEX2* (Bruker, 2005 ▶); cell refinement: *SAINT* (Bruker, 2005 ▶); data reduction: *SAINT*; program(s) used to solve structure: *SHELXS97* (Sheldrick, 2008 ▶); program(s) used to refine structure: *SHELXL97* (Sheldrick, 2008 ▶); molecular graphics: *ORTEP-3 for Windows* (Farrugia, 1997 ▶); software used to prepare material for publication: *SHELXL97* and *WinGX* (Farrugia, 1999 ▶).

## Supplementary Material

Crystal structure: contains datablock(s) I, global. DOI: 10.1107/S1600536812023252/bt5925sup1.cif

Structure factors: contains datablock(s) I. DOI: 10.1107/S1600536812023252/bt5925Isup2.hkl

Supplementary material file. DOI: 10.1107/S1600536812023252/bt5925Isup3.cml

Additional supplementary materials:  crystallographic information; 3D view; checkCIF report

## Figures and Tables

**Table 1:** Hydrogen-bond geometry (Å, °)

*D*—H⋯*A*	*D*—H	H⋯*A*	*D*⋯*A*	*D*—H⋯*A*
C71—H711⋯N4	0.96	2.58	2.900 (2)	100
C72—H721⋯N4	0.96	2.48	2.847 (2)	103
N2—H2⋯N9^i^	0.90 (2)	2.06 (2)	2.9474 (19)	171 (2)

Symmetry code: (i) 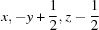.

**Fig. 1.**
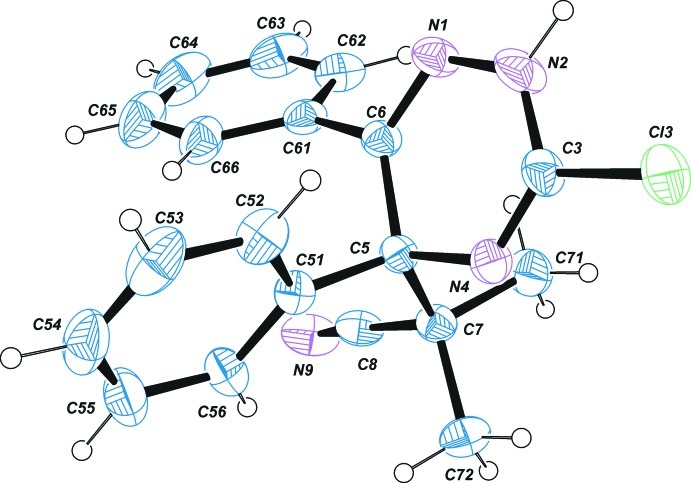
The molecular structure of the title compound, with atom labels and 30% probability displacement ellipsoids for non-H atoms.

**Fig. 2.**
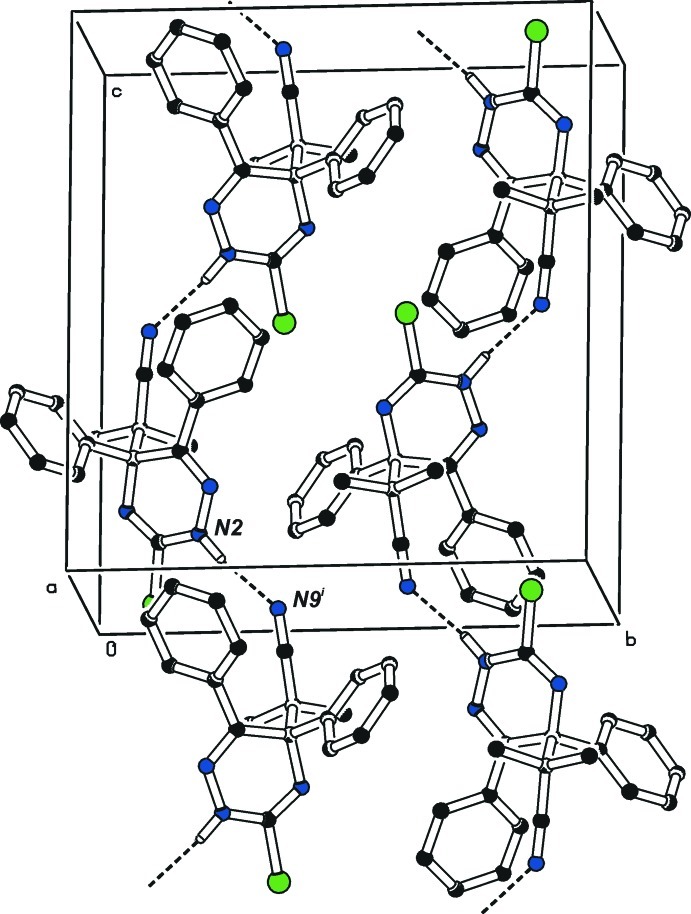
A view of the packing of the title compound. Dashed lines indicate N—H···N intermolecular hydrogen bond [symmetry code: (i) x, –y + 1/2, z–1/2].

## supplementary crystallographic information

### Comment

Compounds containing a chiral oxazoline ring have become the most useful ligand classes for asymmetric catalysis (Hargaden & Guiry, 2009). During our research course on synthesis and application of chiral auxiliaries, synthesis of ligands composed of chiral oxazoline linked with 1,2,4-triazine ring by carbon atom was undertaken. The two step synthetic strategy considered (*a*) nucleophilic substitution of chlorine atom in 3-chloro-5,6-diphenyl-1,2,4-triazine with isobutyronitrile and (*b*) formation of oxazoline ring by condensation of the nitrile group with chiral amino alcohol in the presence of ZnCl_2_ (Coeffard *et al.*, 2009; Fujisawa *et al.*, 1995). In the reaction of 3-chloro-5,6-diphenyl-1,2,4-triazine with isobutyronitrile in the presence of lithium diisopropylamide (LDA) the desired product of chlorine substitution was not formed. Instead of that the title 3-chloro-5-[(1-cyano-1-methyl)ethyl]-5,6-diphenyl-2,5-dihydro-1,2,4-triazine was isolated from the reaction mixture. This unexpected product is a result of covalent addition of isobutyronitrile carbanione to C-5 carbon atom of 1,2,4-triazine ring bearing phenyl substituent. The availability of highly electron-deficient 1,2,4-triazine ring to undergo covalent addition of carbanions at the unsubstituted C-5 carbon is well known (Konno *et al.*, 1987; Rykowski *et al.*, 2000). The result mentioned above is the first example of reaction in which the addition of carbon nucleophlie at C-5, bearing bulky phenyl group, is fully counterbalanced by the high *π*-electron deficiency of the 1,2,4-triazine ring.

The X-ray analysis of (I) undertook in order to confirm its molecular structure and to identification of the proper N2—H/N4—H tautomeric form revealed that this compound exists as N2—H tautomer in the crystalline state. The 2,5-dihydro-1,2,4-triazine ring disubstituted at 5 position is planar to within 0.0089 (13) Å and its geometry is very similar to that observed in related structure of 3-methylthio-2-methyl-5,6-diphenyl-2,5-dihydro-1,2,4-triazine (Ayato *et al.*, 1981). The 5- and 6-phenyl substituents of the 1,2,4-triazine ring are inclined to its mean plane with the dihedral angle of 89.97 (4) and 55.52 (5)°, respectively. The torsion angles N4—C5—C7—C8 = 177.18 (11)°, N4—C5—C7—C71 = -65.69 (14)° and N4—C5—C7—C72 = 55.01 (14)° show that the nitrile and methyl groups of the isopropylcarbonitrile susbstituent adopt the *trans*, *gauche* and *gauche* conformation, respectively, in respect to 1,2,4-triazine ring. In the crystal structure, Fig. 2, the molecules related by a *c* glide plane are linked into chains along the [001] direction by N2—H2···N9 intermolecular hydrogen bond (Table 1).

### Experimental

An oven dried three-necked flask equipped with thermometer was washed with argon and charged with diisopropylamine (0.33 ml, 2.38 mmol) and THF (2 ml). The solution was cooled to -68 °C and butyllithium (1 ml, 2.5 mmol, 1 *M* solution in hexanes) was added trough the septum. The mixture was stirred for 0.5 h. Than, isobutyronitrile (155 mg, 2.25 mmol) was added. After 0.5 h while the carbanione was generated a solution of 3-chloro-5,6-diphenyl-1,2,4-triazine (200 mg, 0.75 mmol) in THF (2 ml) was added dropwise. The mixture was stirred at -68 °C for 1 h, and then wormed to room temperature during 2 h. The reaction was quenched with sat. NH_4_Cl and extracted with ether. The organic layer was dried with MgSO_4_. The solvent was evaporated and the resulting crude product was purified by column chromatography using hexanes/ethyl acetate (5:1) as eluent. The main product was recrystalized from ethanol/water to give 3-chloro-5-[(1-cyano-1-methyl)ethyl]-5,6-diphenyl-2,5-dihydro-1,2,4-triazine, (I), as a colourless crystals; yield: 135 mg, 54%.

### Refinement

All H atom were located by difference Fourier synthesis. The coordinates of the N-bound H atom were refined. H atoms bonded to C were treated as riding on their parent atoms, with C—H distances of 0.93 (aromatic) and 0.96 Å (CH_3_). All H atoms were assigned *U*_iso_(H) values of 1.5*U*_eq_(N,C).

### Crystal data

**Table:**

C_19_H_17_ClN_4_	*F*(000) = 704
*M**_r_* = 336.82	*D*_x_ = 1.264 Mg m^−^^3^
Monoclinic, *P*2_1_/*c*	Melting point: 456 K
Hall symbol: -P 2ybc	Cu *K*α radiation, λ = 1.54178 Å
*a* = 8.2422 (1) Å	Cell parameters from 9905 reflections
*b* = 13.9124 (2) Å	θ = 5.4–67.7°
*c* = 15.4685 (3) Å	µ = 1.96 mm^−^^1^
β = 93.855 (1)°	*T* = 293 K
*V* = 1769.74 (5) Å^3^	Prism, colourless
*Z* = 4	0.44 × 0.23 × 0.11 mm

### Data collection

**Table:**

Bruker SMART APEXII CCD diffractometer	3198 independent reflections
Radiation source: fine-focus sealed tube	2957 reflections with *I* > 2σ(*I*)
Graphite monochromator	*R*_int_ = 0.037
φ and ω scans	θ_max_ = 68.1°, θ_min_ = 4.3°
Absorption correction: multi-scan (*SADABS*; Bruker, 2005)	*h* = −9→9
*T*_min_ = 0.817, *T*_max_ = 1.000	*k* = −12→16
20671 measured reflections	*l* = −18→18

### Refinement

**Table:**

Refinement on *F*^2^	Secondary atom site location: difference Fourier map
Least-squares matrix: full	Hydrogen site location: difference Fourier map
*R*[*F*^2^ > 2σ(*F*^2^)] = 0.037	H atoms treated by a mixture of independent and constrained refinement
*wR*(*F*^2^) = 0.110	*w* = 1/[σ^2^(*F*_o_^2^) + (0.0608*P*)^2^ + 0.3505*P*] where *P* = (*F*_o_^2^ + 2*F*_c_^2^)/3
*S* = 1.03	(Δ/σ)_max_ < 0.001
3198 reflections	Δρ_max_ = 0.21 e Å^−^^3^
221 parameters	Δρ_min_ = −0.28 e Å^−^^3^
0 restraints	Extinction correction: *SHELXL97* (Sheldrick, 2008), Fc^*^=kFc[1+0.001xFc^2^λ^3^/sin(2θ)]^-1/4^
Primary atom site location: structure-invariant direct methods	Extinction coefficient: 0.0014 (3)

### Special details

**Table:**

Experimental. ^1^H NMR (400 MHz, CDCl_3_) *δ*: 1.50 (*s*, 3H, CH_3_), 1.53 (*s*, 3H), 6.95–6.97 (*m*, 2H), 7.21–7.25 (*m*, 2H), 7.34–7.47 (*m*,4H), 7.70–7.72 (*m*, 2H), 8.25 (*s*, 1H); ^13^C NMR (50 MHz, CDCl_3_) *δ*: 22.6, 25.5, 39.0, 70.4, 124.0, 127.9, 128.5, 128.6, 128.7, 129.7, 130.0, 135.4, 140.7, 140.8, 147.5; HR MS ESI calculated for C_19_H_17_N_4_NaCl: 359.10340, found: 359.10469.
Geometry. All esds (except the esd in the dihedral angle between two l.s. planes) are estimated using the full covariance matrix. The cell esds are taken into account individually in the estimation of esds in distances, angles and torsion angles; correlations between esds in cell parameters are only used when they are defined by crystal symmetry. An approximate (isotropic) treatment of cell esds is used for estimating esds involving l.s. planes.
Refinement. Refinement of F^2^ against ALL reflections. The weighted R-factor wR and goodness of fit S are based on F^2^, conventional R-factors R are based on F, with F set to zero for negative F^2^. The threshold expression of F^2^ > 2sigma(F^2^) is used only for calculating R-factors(gt) etc. and is not relevant to the choice of reflections for refinement. R-factors based on F^2^ are statistically about twice as large as those based on F, and R- factors based on ALL data will be even larger.

### Fractional atomic coordinates and isotropic or equivalent isotropic displacement parameters (Å^2^)

**Table:**

	*x*	*y*	*z*	*U*_iso_*/*U*_eq_	
Cl3	0.34703 (6)	0.12753 (4)	0.01120 (3)	0.07817 (19)	
N1	0.63312 (16)	0.25063 (9)	0.18802 (7)	0.0529 (3)	
N2	0.54094 (19)	0.22497 (10)	0.11422 (8)	0.0651 (4)	
H2	0.538 (3)	0.2666 (17)	0.0696 (16)	0.098*	
N4	0.44978 (15)	0.07957 (9)	0.16785 (7)	0.0505 (3)	
N9	0.5113 (2)	0.12362 (10)	0.48082 (9)	0.0693 (4)	
C3	0.45641 (18)	0.14300 (11)	0.11027 (9)	0.0510 (3)	
C5	0.54108 (15)	0.09677 (9)	0.25203 (7)	0.0391 (3)	
C6	0.63422 (15)	0.19302 (9)	0.25246 (8)	0.0425 (3)	
C7	0.40539 (16)	0.10027 (10)	0.31926 (9)	0.0446 (3)	
C8	0.47297 (19)	0.11233 (10)	0.40948 (9)	0.0498 (3)	
C51	0.66526 (15)	0.01512 (9)	0.26417 (8)	0.0432 (3)	
C52	0.77159 (19)	0.00305 (13)	0.19886 (11)	0.0624 (4)	
H52	0.7590	0.0404	0.1490	0.094*	
C53	0.8958 (2)	−0.06387 (16)	0.20727 (16)	0.0840 (6)	
H53	0.9659	−0.0708	0.1630	0.126*	
C54	0.9167 (2)	−0.11954 (14)	0.27919 (19)	0.0848 (7)	
H54	1.0026	−0.1629	0.2853	0.127*	
C55	0.8094 (2)	−0.11095 (13)	0.34268 (15)	0.0773 (6)	
H55	0.8205	−0.1506	0.3911	0.116*	
C56	0.6847 (2)	−0.04408 (11)	0.33588 (10)	0.0562 (4)	
H56	0.6136	−0.0390	0.3798	0.084*	
C61	0.72769 (17)	0.22915 (10)	0.33162 (8)	0.0489 (3)	
C62	0.6891 (2)	0.31924 (12)	0.36333 (10)	0.0610 (4)	
H62	0.6081	0.3557	0.3342	0.091*	
C63	0.7696 (3)	0.35509 (16)	0.43739 (13)	0.0800 (6)	
H63	0.7418	0.4152	0.4582	0.120*	
C64	0.8905 (3)	0.3025 (2)	0.48049 (13)	0.0919 (7)	
H64	0.9435	0.3263	0.5310	0.138*	
C65	0.9325 (2)	0.21470 (19)	0.44865 (13)	0.0859 (6)	
H65	1.0160	0.1797	0.4772	0.129*	
C66	0.85223 (19)	0.17737 (14)	0.37446 (11)	0.0648 (4)	
H66	0.8819	0.1177	0.3535	0.097*	
C71	0.2966 (2)	0.18857 (13)	0.29982 (13)	0.0668 (4)	
H711	0.2400	0.1813	0.2439	0.100*	
H712	0.3627	0.2454	0.3002	0.100*	
H713	0.2192	0.1941	0.3433	0.100*	
C72	0.29850 (19)	0.00935 (12)	0.31542 (11)	0.0597 (4)	
H721	0.2496	0.0014	0.2578	0.090*	
H722	0.2148	0.0157	0.3553	0.090*	
H723	0.3644	−0.0457	0.3308	0.090*	

### Atomic displacement parameters (Å^2^)

**Table:**

	*U*^11^	*U*^22^	*U*^33^	*U*^12^	*U*^13^	*U*^23^
Cl3	0.0896 (3)	0.0984 (4)	0.0432 (2)	−0.0087 (2)	−0.0200 (2)	0.01157 (19)
N1	0.0687 (7)	0.0523 (7)	0.0379 (6)	−0.0075 (6)	0.0044 (5)	0.0062 (5)
N2	0.0957 (10)	0.0610 (8)	0.0373 (6)	−0.0108 (7)	−0.0056 (6)	0.0144 (6)
N4	0.0542 (6)	0.0599 (7)	0.0363 (6)	−0.0071 (5)	−0.0053 (5)	0.0048 (5)
N9	0.1083 (11)	0.0611 (8)	0.0406 (8)	−0.0151 (7)	0.0214 (7)	−0.0087 (6)
C3	0.0568 (8)	0.0615 (8)	0.0342 (7)	0.0031 (6)	−0.0006 (6)	0.0045 (6)
C5	0.0436 (6)	0.0443 (7)	0.0294 (6)	−0.0012 (5)	0.0020 (5)	0.0027 (5)
C6	0.0458 (7)	0.0463 (7)	0.0359 (6)	−0.0019 (5)	0.0062 (5)	0.0035 (5)
C7	0.0468 (7)	0.0452 (7)	0.0427 (7)	0.0002 (5)	0.0105 (5)	0.0017 (5)
C8	0.0669 (9)	0.0416 (7)	0.0431 (8)	−0.0056 (6)	0.0199 (6)	−0.0025 (5)
C51	0.0455 (7)	0.0453 (7)	0.0384 (6)	0.0009 (5)	0.0006 (5)	−0.0068 (5)
C52	0.0567 (8)	0.0755 (10)	0.0561 (9)	0.0013 (7)	0.0128 (7)	−0.0147 (8)
C53	0.0544 (9)	0.0908 (14)	0.1085 (17)	0.0050 (9)	0.0188 (10)	−0.0426 (13)
C54	0.0555 (10)	0.0640 (11)	0.132 (2)	0.0144 (8)	−0.0152 (11)	−0.0275 (12)
C55	0.0779 (12)	0.0562 (9)	0.0933 (14)	0.0143 (8)	−0.0274 (11)	−0.0015 (9)
C56	0.0652 (9)	0.0516 (8)	0.0507 (8)	0.0089 (7)	−0.0041 (6)	0.0021 (6)
C61	0.0508 (7)	0.0578 (8)	0.0384 (7)	−0.0150 (6)	0.0063 (5)	0.0044 (6)
C62	0.0713 (10)	0.0604 (9)	0.0523 (8)	−0.0177 (7)	0.0117 (7)	−0.0040 (7)
C63	0.0877 (13)	0.0883 (13)	0.0657 (11)	−0.0331 (11)	0.0168 (10)	−0.0239 (10)
C64	0.0823 (13)	0.137 (2)	0.0561 (10)	−0.0389 (14)	0.0017 (9)	−0.0277 (12)
C65	0.0641 (10)	0.1296 (18)	0.0616 (11)	−0.0149 (11)	−0.0145 (8)	−0.0034 (12)
C66	0.0542 (8)	0.0840 (11)	0.0551 (9)	−0.0092 (8)	−0.0050 (7)	−0.0005 (8)
C71	0.0556 (9)	0.0645 (10)	0.0818 (11)	0.0150 (7)	0.0167 (8)	0.0069 (8)
C72	0.0584 (8)	0.0620 (9)	0.0602 (9)	−0.0148 (7)	0.0150 (7)	−0.0023 (7)

### Geometric parameters (Å, º)

**Table:**

Cl3—C3	1.7383 (14)	C54—H54	0.9300
N1—C6	1.2787 (17)	C55—C56	1.385 (2)
N1—N2	1.3752 (18)	C55—H55	0.9300
N2—C3	1.336 (2)	C56—H56	0.9300
N2—H2	0.90 (2)	C61—C66	1.386 (2)
N4—C3	1.2577 (18)	C61—C62	1.390 (2)
N4—C5	1.4790 (16)	C62—C63	1.378 (2)
N9—C8	1.138 (2)	C62—H62	0.9300
C5—C51	1.5321 (18)	C63—C64	1.372 (3)
C5—C6	1.5433 (18)	C63—H63	0.9300
C5—C7	1.5782 (17)	C64—C65	1.371 (4)
C6—C61	1.4895 (18)	C64—H64	0.9300
C7—C8	1.477 (2)	C65—C66	1.387 (2)
C7—C71	1.539 (2)	C65—H65	0.9300
C7—C72	1.5403 (19)	C66—H66	0.9300
C51—C56	1.382 (2)	C71—H711	0.9600
C51—C52	1.3914 (19)	C71—H712	0.9600
C52—C53	1.383 (3)	C71—H713	0.9600
C52—H52	0.9300	C72—H721	0.9600
C53—C54	1.357 (3)	C72—H722	0.9600
C53—H53	0.9300	C72—H723	0.9600
C54—C55	1.370 (3)		
			
C6—N1—N2	117.32 (12)	C54—C55—C56	121.02 (19)
C3—N2—N1	121.13 (12)	C54—C55—H55	119.5
C3—N2—H2	121.8 (15)	C56—C55—H55	119.5
N1—N2—H2	117.0 (15)	C51—C56—C55	120.44 (16)
C3—N4—C5	117.79 (12)	C51—C56—H56	119.8
N4—C3—N2	127.89 (14)	C55—C56—H56	119.8
N4—C3—Cl3	119.52 (12)	C66—C61—C62	118.65 (15)
N2—C3—Cl3	112.59 (10)	C66—C61—C6	122.90 (14)
N4—C5—C51	106.50 (10)	C62—C61—C6	118.45 (14)
N4—C5—C6	111.57 (10)	C63—C62—C61	120.73 (18)
C51—C5—C6	108.35 (10)	C63—C62—H62	119.6
N4—C5—C7	104.12 (10)	C61—C62—H62	119.6
C51—C5—C7	116.06 (10)	C64—C63—C62	120.3 (2)
C6—C5—C7	110.17 (10)	C64—C63—H63	119.9
N1—C6—C61	113.92 (12)	C62—C63—H63	119.9
N1—C6—C5	124.27 (12)	C65—C64—C63	119.56 (18)
C61—C6—C5	121.74 (10)	C65—C64—H64	120.2
C8—C7—C71	105.76 (12)	C63—C64—H64	120.2
C8—C7—C72	107.95 (11)	C64—C65—C66	120.9 (2)
C71—C7—C72	108.90 (12)	C64—C65—H65	119.6
C8—C7—C5	112.78 (11)	C66—C65—H65	119.6
C71—C7—C5	108.99 (11)	C61—C66—C65	119.87 (19)
C72—C7—C5	112.22 (11)	C61—C66—H66	120.1
N9—C8—C7	173.86 (17)	C65—C66—H66	120.1
C56—C51—C52	117.77 (14)	C7—C71—H711	109.5
C56—C51—C5	125.50 (12)	C7—C71—H712	109.5
C52—C51—C5	116.66 (13)	H711—C71—H712	109.5
C53—C52—C51	120.74 (18)	C7—C71—H713	109.5
C53—C52—H52	119.6	H711—C71—H713	109.5
C51—C52—H52	119.6	H712—C71—H713	109.5
C54—C53—C52	120.91 (18)	C7—C72—H721	109.5
C54—C53—H53	119.5	C7—C72—H722	109.5
C52—C53—H53	119.5	H721—C72—H722	109.5
C53—C54—C55	119.04 (16)	C7—C72—H723	109.5
C53—C54—H54	120.5	H721—C72—H723	109.5
C55—C54—H54	120.5	H722—C72—H723	109.5
			
C6—N1—N2—C3	−0.1 (2)	C6—C5—C51—C56	112.84 (14)
C5—N4—C3—N2	2.1 (2)	C7—C5—C51—C56	−11.66 (19)
C5—N4—C3—Cl3	−178.37 (10)	N4—C5—C51—C52	56.07 (15)
N1—N2—C3—N4	−1.5 (3)	C6—C5—C51—C52	−64.08 (15)
N1—N2—C3—Cl3	178.95 (12)	C7—C5—C51—C52	171.42 (12)
C3—N4—C5—C51	−119.22 (14)	C56—C51—C52—C53	−2.2 (2)
C3—N4—C5—C6	−1.18 (17)	C5—C51—C52—C53	175.00 (15)
C3—N4—C5—C7	117.62 (13)	C51—C52—C53—C54	0.2 (3)
N2—N1—C6—C61	−176.07 (12)	C52—C53—C54—C55	2.2 (3)
N2—N1—C6—C5	0.8 (2)	C53—C54—C55—C56	−2.5 (3)
N4—C5—C6—N1	−0.21 (18)	C52—C51—C56—C55	1.8 (2)
C51—C5—C6—N1	116.72 (14)	C5—C51—C56—C55	−175.06 (14)
C7—C5—C6—N1	−115.34 (14)	C54—C55—C56—C51	0.5 (3)
N4—C5—C6—C61	176.45 (12)	N1—C6—C61—C66	−125.41 (15)
C51—C5—C6—C61	−66.62 (14)	C5—C6—C61—C66	57.61 (18)
C7—C5—C6—C61	61.32 (15)	N1—C6—C61—C62	53.96 (17)
N4—C5—C7—C8	177.18 (11)	C5—C6—C61—C62	−123.02 (14)
C51—C5—C7—C8	60.49 (15)	C66—C61—C62—C63	−2.0 (2)
C6—C5—C7—C8	−63.07 (14)	C6—C61—C62—C63	178.61 (14)
N4—C5—C7—C71	−65.69 (14)	C61—C62—C63—C64	0.7 (3)
C51—C5—C7—C71	177.63 (12)	C62—C63—C64—C65	1.0 (3)
C6—C5—C7—C71	54.07 (14)	C63—C64—C65—C66	−1.3 (3)
N4—C5—C7—C72	55.01 (14)	C62—C61—C66—C65	1.7 (2)
C51—C5—C7—C72	−61.68 (15)	C6—C61—C66—C65	−178.98 (15)
C6—C5—C7—C72	174.76 (11)	C64—C65—C66—C61	0.0 (3)
N4—C5—C51—C56	−127.01 (14)		

### Hydrogen-bond geometry (Å, º)

**Table:**

*D*—H···*A*	*D*—H	H···*A*	*D*···*A*	*D*—H···*A*
C71—H711···N4	0.96	2.58	2.900 (2)	100
C72—H721···N4	0.96	2.48	2.847 (2)	103
N2—H2···N9^i^	0.90 (2)	2.06 (2)	2.9474 (19)	171 (2)

Symmetry code: (i) *x*, −*y*+1/2, *z*−1/2.
